# Infection, inflammation and colon carcinogenesis

**DOI:** 10.18632/oncotarget.624

**Published:** 2012-08-19

**Authors:** G.N. Wogan, P.C. Dedon, S.R. Tannenbaum, J.G. Fox

**Affiliations:** Department of Biological Engineering and Department of Chemistry, MIT, Cambridge, MA; Department of Biological Engineering, MIT, Cambridge, MA; Division of Comparative Medicine and Department of Biological Engineering, MIT, Cambridge MA; Department of Biological Engineering and Department of Chemistry, MIT, Cambridge, MA

The importance of chronic inflammation as a risk factor for major cancers is well documented [1], and the inflammatory state is known to involve contributions of both adaptive and innate immune components. In a recent publication [2] we describe an experimental animal model in which infection, inflammation and cancer are mechanistically linked, and provide evidence that chemical mediators of the innate immune system and bacterial toxins both play key roles in driving colon carcinogenesis. In this model, epithelial injury caused by *Helicobacter hepaticus* infection enhances access of bacterially-associated products to pattern-recognition receptors located on surfaces of macrophages and dendritic cells. Receptor ligation leads to activation of transcription factors, including NF-kappa B, that regulate production of chemo-attractants for macrophages and neutrophils, recruitment of which is a hallmark of inflammation. These acute inflammatory events are re-enforced by expression of powerful inflammatory mediators such as TNF-α and IL-2, which amplify acute inflammatory gene expression and enhance cell survival. If not properly extinguished, the innate inflammatory response is maintained and further amplified by activation of cell-mediated adaptive immunity.

There is now strong evidence that biomolecular damage found in inflamed tissues is caused by a battery of highly reactive oxygen and nitrogen species capable of damaging all classes of biomolecules. Reactive species are generated mainly by activated macrophages and neutrophils, but can also be produced at functionally significant levels endogenously within epithelial cells themselves [3, 4]. In an earlier investigation [5], we identified the critical importance of NO and neutrophils in the carcinogenic process, and in our recent study [2] we demonstrated that neutrophils contribute to DNA damage through myeloperoxidase-driven formation of hypochlorous acid and the subsequent formation of 5-chlorodeoxycytosine. These chemicals and reaction products derived from them cause injury, death and mutation to cells in their immediate environment, the ultimate result being intensification of damage inflicted by the inflammatory process.

What we have also learned from recent studies is that cell death and genotoxicity in this environment are not induced solely by chemicals arising from immune cells or epithelial cells, but that these effects are augmented by bacterial toxins (e.g., cytolethal distending toxin in *H. hepaticus*) that cause DNA strand breaks, inhibit ATM-dependent response pathways, and suppress repair of DNA adducts [6, 7]. Concurrence of the two mechanisms of DNA damage and compensatory increases in cell replication creates a perfect storm of conditions enhancing the probability of neoplastic transformation.

Inflammation associated with infections can occur over protracted periods of time. DNA damage induced as a result of inflammation is potently mutagenic, and infection followed by inflammation is a biologically plausible scenario that could explain the abundant mutations observed in some human tumors. Inflammatory bowel disease (IBD), which is known to be associated with increased cancer risk, is particularly relevant to our continuing efforts to elucidate mechanisms underlying these interactions. IBD results from intermittent and severe activation of the mucosal immune system in the gastrointestinal tract to promote chronic inflammation. Infiltration of gut tissue by lymphocytes, neutrophils, and macrophages results in prolonged exposure to pro-inflammatory cytokines and to highly reactive chemical species that induce oxidation, nitration and chlorination of DNA, RNA and proteins. Secondary effects also result from oxidation of unsaturated lipids, creating a cascade of highly reactive unsaturated carbonyls, which also damage DNA, RNA, and proteins. Chronic exposure to products of inflammation can thus result in chemical damage to all classes of cellular macromolecules, altered protein expression, and dysregulated cell proliferation.

Current theories cite the intestinal microbiome in IBC as a central driver of both inflammation and subsequent development of dysplasia in genetically-predisposed individuals, acting similarly in experimental models involving mice with immune dysregulation. These processes appear likely to be associated with the pathogenesis of cancer development. Interestingly, other investigators recently identified a genetic locus, *Hiccs*, part of a 1.71-Mb interval on chromosome 3, as a major susceptibility locus for *H. hepaticus*-induced colitis and colon cancer in *H. hepaticus*-infected 129 Rag−/− mice also treated with azoxymethane [8]. This locus controls induction of the innate inflammatory response by regulating cytokine production and granulocyte recruitment by Thy1^+^ innate lymphoid cells. Analogous pathways may be operable in IBD and associated colorectal cancers in humans.

While alterations of the intestinal microbiome have been described in IBD patients, with suggestions that particular species may be associated with ileal Crohn's disease, no individual bacterial species or groups have been consistently associated with either colonic Crohn's disease or ulcerative colitis. Likewise, the role that microbial biomolecular activity may play in these diseases remains unknown. Metabolomic analysis of fecal water from patients with these diseases has identified microbial population shifts suggesting that functional capacity may be more critical than microbial membership [9]. While chronic inflammation is widely thought to be a critical factor, biomolecular pathways implicated in the development of IBD-associated colon cancer remain incompletely characterized.
